# Predicting Age From Brain EEG Signals—A Machine Learning Approach

**DOI:** 10.3389/fnagi.2018.00184

**Published:** 2018-07-02

**Authors:** Obada Al Zoubi, Chung Ki Wong, Rayus T. Kuplicki, Hung-wen Yeh, Ahmad Mayeli, Hazem Refai, Martin Paulus, Jerzy Bodurka

**Affiliations:** ^1^Laureate Institute for Brain Research, Tulsa, OK, United States; ^2^Department of Electrical and Computer Engineering, University of Oklahoma, Tulsa, OK, United States; ^3^Stephenson School of Biomedical Engineering, University of Oklahoma, Norman, OK, United States

**Keywords:** aging, human brain, EEG, machine learning, feature extraction, BrainAGE

## Abstract

**Objective:** The brain age gap estimate (BrainAGE) is the difference between the estimated age and the individual chronological age. BrainAGE was studied primarily using MRI techniques. EEG signals in combination with machine learning (ML) approaches were not commonly used for the human age prediction, and BrainAGE. We investigated whether age-related changes are affecting brain EEG signals, and whether we can predict the chronological age and obtain BrainAGE estimates using a rigorous ML framework with a novel and extensive EEG features extraction.

**Methods:** EEG data were obtained from 468 healthy, mood/anxiety, eating and substance use disorder participants (297 females) from the Tulsa-1000, a naturalistic longitudinal study based on Research Domain Criteria framework. Five sets of preprocessed EEG features across channels and frequency bands were used with different ML methods to predict age. Using a nested-cross-validation (NCV) approach and stack-ensemble learning from EEG features, the predicted age was estimated. The important features and their spatial distributions were deduced.

**Results:** The stack-ensemble age prediction model achieved *R*^2^ = 0.37 (0.06), Mean Absolute Error (MAE) = 6.87(0.69) and RMSE = 8.46(0.59) in years. The age and predicted age correlation was *r* = 0.6. The feature importance revealed that age predictors are spread out across different feature types. The NCV approach produced a reliable age estimation, with features consistent behavior across different folds.

**Conclusion:** Our rigorous ML framework and extensive EEG signal features allow a reliable estimation of chronological age, and BrainAGE. This general framework can be extended to test EEG association with and to predict/study other physiological relevant responses.

## Introduction

Brain changes due to age have been studied for decades (e.g., Lindsley, 1939; Harmony et al., 1990; Lao et al., 2004) and more recently using genetics Lu et al. (2017). The term BrainAGE (the difference between predicted age—chronological age) was introduced to examine and capture any disease-related deviations from natural aging, by comapring BrainAGE estimates in disease group to healthy control group. Magnetic Resonance Imaging (MRI) has been widely used to build predictive models for age by utilizing white matter (WM) and gray matter (GM) properties. Franke et al. (2010) employed T1-weighted (T1w) MRI structural images to establish a framework (using a kernel method for regression) for automatically and efficiently estimating the age of healthy individuals. This framework proved to be a reliable, scanner-independent, and efficient method for age estimation in healthy subjects, yielding a correlation of *r* = 0.92 between the estimated and the real age in the test samples and a mean absolute error of 5 years. Similarly, Cole et al. (2017) used Deep Learning (DL) to study BrainAGE using both pre-processed and raw T1w MRI images. Their approach predicted age with minimal efforts by achieving a correlation between age and predicted-age of *r* = 0.96 and MAE = 4.16 years. Also, Valizadeh et al. (2017) obtained *R*^2^ = 0.77 from large healthy subjects (*n* = 3,144) by training features from various anatomical brain regions. Càmara et al. (2007) studied age-related changes in water self-diffusion in cerebral white matter using Diffusion Tensor Imaging (DTI). Their results revealed white matter changes with age in different brain regions, like the corpus callosum, prefrontal regions, the internal capsule, the hippocampal complex, and the putamen. Functional MRI (fMRI) imaging was also used to predict age alone, or combined with other imaging approach. For instance, Dosenbach et al. (2010) were able to explain up to 55% of their sample variance from the functional MRI connectivity (fcMRI) data. Likewise, Qin et al. (2015) related the developmental changes in the amplitude of low-frequency spontaneous fluctuations in resting-state fMRI to age. They reported MAE of 4.6 years between chronological age and predicted-age. More recently, Liem et al. (2017) utilized cortical anatomy and whole-brain functional connectivity for predicting brain-based age achieving MAE = 4.29 year. Several BrainAGE studies revealed changes and differences among clinical groups. For example, BrainAGE estimations in schizophrenia patients was attributed to accelerated aging when compared to healthy and bipolar subjects (Nenadić et al., 2017). In additions, individuals diagnosed with medically refractory epilepsy had a higher predicted age than health subjects (Pardoe et al., 2017).

Herein, we focus on studying BrainAGE using EEG signals. Several studies have demonstrated that EEG features like EEG rhythmic activity (e.g., delta, theta, alpha, beta, and gamma) changes as a function of age (Matthis et al., 1980; Clarke et al., 2001; Marshall et al., 2002; Ashburner, 2007; Cragg et al., 2011). For instance, Benninger et al. (1984) found theta band showed an increase in power spectra, while delta exhibited decrease for healthy children between 4 and 17 years. Gasser et al. (1988) showed that: (i) the relative power increases with age in fast bands, while decreases for the slow bands in healthy children and adolescent (6–17 years), (ii) all bands showed increase in the absolute power except for alpha-2. Analyzing the coherence of EEG in resting state revealed that younger healthy subjects had a lower coherence than elderly ones for theta, alpha-3, beta-2, and beta-2 (Kikuchi et al., 2000). The beta relative power was positively correlated with age for older subjects for resting with eye closed condition (Marciani et al., 1994). The alpha reactivity decreased and showed negative correlation with age in the older group when performing mental tasks (Marciani et al., 1994). The theta power was shown to increase from resting to arithmetic task for younger group, while decreasing for the older group (Widagdo et al., 1998). Moreover, the delta band beta-3 power showed an increase from resting to arithmetic tasks, while alpha was decreased (Widagdo et al., 1998). A more recent study used four channels EEG recording to investigate age-related changes in EEG power from thousands of subjects throughout adulthood (Hashemi et al., 2016). Their findings showed an overall age-related shift in band power from lower to a higher frequency and a gradual slowing of the peak α frequency with age. Furthermore, studying the source of cortical rhythm suggested that occipital delta and posterior cortical alpha rhythms decrease in magnitude during physiological aging with both linear and nonlinear trends (Babiloni et al., 2006). Age prediction from EEG was studied in Dimitriadis and Salis (2017), where authors used functional connectivity features from EEG to predict age from 94 healthy subjects. Their results showed accuracy of *R*^2^ = 0.60 for eyes-open and *R*^2^ = 0.48 for eyes-closed.

The influence of diseases on EEG features were investigated elsewhere. For instance, Saletu et al. (1995) used the mean EEG power spectrum to study group differences between multi-infract dementia (MID) and dementia of Alzheimer's disease (AD) and compared it a healthy group. MID group showed a significant increase of theta activity in occipital regions and decrease in alpha activity. Abnormalities in cortical neural synchronization for subjects were observed in subjects with mild cognitive impairment due to AD (ADMCI) and to Parkinson Disease (PDMCI) in delta and alpha (Babiloni et al., 2016). Differentiating subjects with Alzheimer's disease from healthy ones was studied in Babiloni et al. (2016). Authors reported 70% accuracy using the power and functional connectivity of cortical sources, which was later improved to 77% using Artificial Neural Network (Triggiani et al., 2017). Table 1 provides a summary of studies that specifically reported age prediction performance from brain imaging data.

**Table 1:** A summary of related work for predicting age from brain imaging data.

**Work**	**Data**	**No. of Samples**	**Performance**
Franke et al., 2010	MRI	650	*r* = 0.92, MAE = 5 years
Cole et al., 2017	MRI	2,001	*r* = 0.96, MAE = 4.16 years
Dosenbach et al., 2010	fMRI	238	*R*^2^ = 0.55
Qin et al., 2015	fMRI	183	MAE = 4.6 years
Valizadeh et al., 2017	MRI	3,144	*R*^2^ = 0.77
Dimitriadis and Salis, 2017	EEG	94	*R*^2^ = 0.6 for eyes open *R*^2^ = 0.48 for eyes closed
Liem et al., 2017	fMRI + MRI	2,354	MAE = 4.29 years

In this study, we proposed a robust and rigorous framework to predict BrainAGE using different features of EEG signals recorded during fMRI. First, we extended a recent open-source EEG feature extraction software in Matlab (Toole and Boylan, 2017) to provide a feature representation of individual subjects. Then, we applied a set of machine learning (ML) methods to predict age from features. Our proposed framework and a proof-of-concept analysis revealed that robust BrainAGE predictors span multiple EEG signal features, including separate channels, and frequencies. The overall accuracy elaborated that EEG BrainAGE is a promising approach to study brain maturity and has capacity to reveal different factors that affect natural aging.

## Methods

### Participants

Participants were selected from the first 500 subjects of the Tulsa 1000 (T-1000), a naturalistic study that is assessing and longitudinally following 1,000 individuals, including healthy comparisons and treatment-seeking individuals with mood disorders and/or anxiety, substance use, and eating disorders. The T-1000 aims to determine how disorders of affect, substance use, and eating behavior organize across different levels of analysis with a focus on predictors of long-term prognosis, symptom severity, and treatment outcome (Victor et al., 2018). The T-1000 study is conducted at the Laureate Institute for Brain Research. The study human research protocol was approved by the Western Institutional Review Board. All participants provided written informed consent and received financial compensation for participation. As described in details in Victor et al. (2018), the study participants were screened on the basis of a treatment-seeking history and dimensional psychopathology scores: Patient Health Questionnaire (PHQ-9) ≥ 10 and/or Overall Anxiety Severity and Impairment Scale (OASIS) ≥ 8, Drug Abuse Screening Test (DAST-10) score > 3, or Eating Disorder Screen (SCOFF) score ≥ 2. Each participant underwent approximately 24 h of testing over the course of 1 year including a standardized diagnostic assessment, self-report questionnaires, behavioral and physiological measurements indexing RDoC domains, magnetic resonance imaging focusing on brain structure and reward-related processing, fear processing, cognitive control/inhibition, interoceptive processing, and blood/microbiome collection. Please refer to Figure S1 in Supplementary for the detailed information about the demographics of the dataset.

### EEG recording

EEG signals were recorded simultaneously with fMRI using a 32-channel MR-compatible EEG system arranged according to the international 10–20 system from Brain Products GmbH. ECG signal was recorded using an electrode on the subject's back. In order to synchronize the EEG system clock with the 10 MHz MRI scanner clock, a Brain Products' SyncBox device was utilized. The EEG acquisition temporal resolution, and measurement resolution were 0.2 ms (i.e., 16-bit 5 kS/s sampling) and 0.1 μV respectively. A hardware filtering throughout the acquisition in a frequency band between 0.016 and 250 Hz was applied to EEG signals.

We included EEG data collected from 468 subjects (mean age: 34.8 years, 297 females). One resting EEG-fMRI run was conducted for each subject; lasting 8 min. The participants were instructed to relax and keep their eyes open and fixate on a cross.

Magnetic resonance (MR) images were acquired simultaneously via a General Electric Discovery MR750 whole-body 3 T MRI scanner with a standard 8-channel, receive-only head coil array. A single-shot gradient-recalled EPI sequence with Sensitivity Encoding (SENSE) was employed for the fMRI acquisition. The fMRI data has not been used in this paper.

### EEG data preprocessing

For each scan the EEG data was preprocessed with an in-house script developed in MATLAB. The script was designed to remove the MR gradient artifact and cardioballistic artifact from the EEG data. The details about the preprocessing script are given as follow. The MR gradient artifact was first removed from the EEG data using optimal basis sets (Allen et al., 2000; Delorme and Makeig, 2004; Niazy et al., 2005). Then the EEG data was band-pass filtered between 1 and 70 Hz, down-sampled to 4 ms temporal resolution, and band-stop filtered (1 Hz bandwidth) at the harmonics of the fMRI slice selection frequency (19.5 Hz), AC power line frequency (60 Hz), and a 26 Hz vibration artifact frequency (Mayeli et al., 2016). Then the cardioballistic artifact was corrected using optimal basis sets subtraction (Niazy et al., 2005), which requires the timing of the artifact cycle. In order to achieve a robust artifact cycle determination, the script determined the artifact cycle using the cardioballistic component directly from the EEG-fMRI data (Wong et al., 2018), which was extracted by independent component analysis (Bell and Sejnowski, 1995) and was automatically identified (Wong et al., 2016).

### EEG feature extraction

Feature extraction is a quintessential phase in any EEG analysis that depends on finding common features representation among EEG samples. The existing literature provides quite extensive span of features extraction using variety of signal processing approaches (Jenke et al., 2014). Choosing feature extraction method relies on the applications of the prediction and the compromisation between interpretation and performance. For instance, advanced features extraction methods can be used at the cost of interpretation, where such approaches have been shown to outperform the typical approaches (Dimitriadis and Salis, 2017; Al Zoubi et al., 2018). In our case, BrainAGE emphasizes on the interpretation and understanding of the predictors since the goal is to find those features that influence BrainAGE modeling. Thus, we adopted the similar set of features used by Toole and Boylan (2017), which extracts a wide range of commonly used features from EEG. However, our work takes an extensive approach to survey all features from all channels and bands without reducing features by averaging as done in Toole and Boylan (2017). That is, all features from all possible channels, bands and across different types of features were extracted from EEG. In addition, the types of features used here are commonly used in literature to analyze EEG data. That is, the interpretation and replication of such features are less challenging than using uncommon features. However, our approach results in a relatively large number of features from EEG. Therefore, a feature selection and suitable ML algorithms are needed to deduce the important predictors. All features were extracted from each subject independently and arranged in one row/sample.

#### General configuration

**Figure 1:**
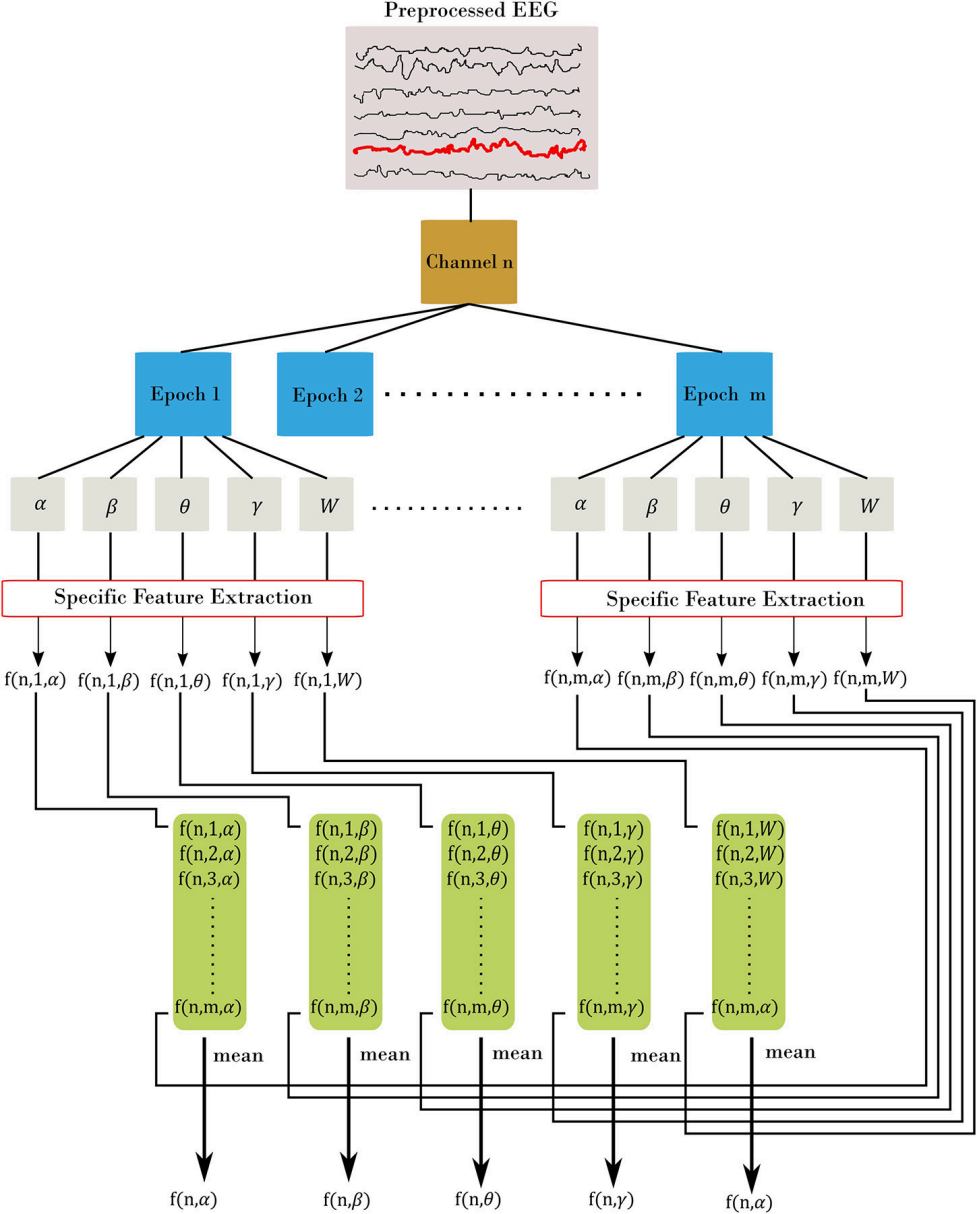
Feature extraction procedure. Each channel is divided into m epoch. From there, we filtered each epoch into α, β, θ, γ, and W frequency bands. Then, for each filtered epoch, we applied the desired feature. This resulted in m feature value from all epochs, which are then averaged to estimate the channel-level feature. In the figure, we represent each feature using three indices: f(channel, epoch, band) with channel = [1.N], epoch = [1.m], and band = [α, β, θ, γ, W]. The final out is a channel-level feature and represented with two indices f(channel, band).

EEG bands of interest are [δ = 0.5–4; θ = 4–7; α = 7–13; β = 13–30; *W* = 0.5–30] Hz using the bipolar montage of the EEG, with W denotes the whole frequency range of EEG. We denoted EEG time series as *x*_*i*_[*n*] with frequency bands of *i* = α, β, θ, γ, *W* and *n* is channel's index (the total number of channels is *N* = 31). We selected five types of features as follows: amplitude, range, spectral, connectivity, and fractal dimension. We divided the EEG recordings from each subject into 60 s and 50% overlap among epochs (14 epochs). Figure 1 elaborates on the features extraction process. For each channel, we divided the signal into m epochs, then we filtered each epoch into corresponding frequency bands. A specific feature extraction was applied to each sub-segment yielding *m* values. Finally, we estimated the channel-level feature for the corresponding frequency band as the average across all epochs. The process is slightly different for Fractal Dimension (FD) features, since we estimate the features without filtering into the frequency bands.

#### Amplitude domain features

The amplitude features characterize the statistical properties of the signal power $A_{\text{power}}^{i}$ and the signal envelope $E_{\text{mean}}^{i}$. We calculated: (i) the mean, (ii) standard deviation, (iii) skewness, and (iv) kurtosis for each channel across frequency bands. The $E_{\text{mean}}^{i}\;$is calculated using mean of the envelop *e*[*n*]_*i*_, which is identified in complex notation as:${\;e}_{i}\left[n\right]={\left|x_{i}\left[n\right]+\mathrm{jH}\left\{x_{i}\left[n\right]\right\}\right|}^{2}$, with which is the Hilbert transformation.

#### Range domain features (rEEG)

Range features account for the peak-to-peak voltages changes and characterize changes in the signal over the time. To achieve that, we segmented each epoch into short-time portions each with a window size of *w* = 2 *s* and overlap = 50%. Then, for each segment, we calculated the corresponding range of peak-to-peak. This produced samples from each epoch to estimate the mean, median, 5th and 95th percentiles, standard deviation, coefficient of variation and the measure of symmetry.

#### Spectral domain features

Spectral features have been the most commonly used features for EEG. To extract the spectral features, we applied Welch periodgram to estimate the power spectral density (PSD) and the hamming window with a length of 2 s and overlap of 50%. The following spectral features have been extracted: (1) power, (2) relative power, (3) entropy (using Wiener and Shannon methods), (4) edge frequency (the cut-off frequency at which encompasses 95% of spectral power), and (5) differences between consecutive short-time spectral estimations.

#### Connectivity domain features

We calculated the brain symmetry index (BSI) as the mean of PSD difference between the left and right hemispheres for each frequency band (*K* = δ, θ, α, β, γ).

Let *a*_*i*_ and *b*_*i*_ be the lower and upper frequency limit of band *i*, the BSI for band *i* is:

(1) $$\begin{array}{l} C_{BSI}^{i}=\frac{1}{\left(b_{i}-a_{i}\right)}\sum_{k=a_{i}}^{b_{i}}\left|\frac{P_{left}\left[K\right]\;-\;P_{right}\left[K\right]}{P_{left}\left[K\right]\;+\;P_{right}\left[K\right]}\right| \end{array}$$

With

(2) $$\begin{array}{l} P_{\text{left}}\left[K\right]=\frac{\sum_{m=1}^{n/2}P_{m}\left[K\right]}{n/2}\text{and }P_{\text{right}}\left[K\right]=\frac{\sum_{m=\frac{M}{2}+1}^{M}P_{m}\left[K\right]}{n/2} \end{array}$$

Also, we calculated the median and lag of maximum correlation coefficient of the Spearman correlation between envelopes of hemisphere-paired channels and coherence between channel pairs.

#### Fractal dimension domain features

Fractal dimension for time series is a value that estimates to what extent the fractal pattern changes with respect to the scale at which it embeds. We applied Higuchi method with *k* = 6 for each EEG channel to estimate the FD.

Table 2 summarizes the extracted set of features from EEG data.

**Table 2:** The extracted features from EEG data.

**Feature group**	**Subset of features**	**Across bands**	**Across channels**	**Number of features**
Amplitude	Total power, mean, standard deviation, skewness, kurtosis, envelope mean, and standard deviation	Yes	Yes	6 × 4 × 31
Peak-to-peak	Mean, median, 5th and 95th percentiles, standard deviation, the coefficient of variation and the measure of symmetry	Yes	Yes	7 × 4 × 31
Spectral power	Spectral power and relative power, spectral entropy (using Wiener and Shannon methods), spectral edge frequency (the cut-off frequency at which encompasses 95% of spectral power) and spectral differences between consecutive short-time spectral estimations	Yes	Yes	6 × 4 × 31
Connectivity	Brain symmetry index, correlation, mean and maximum of frequency at which the maximum coherence is achieved	Yes	No	5 × 4
Fractal dimension	Fractal dimension	No	Yes	31

### Feature reduction

After feature extraction, we eliminated features that are either low in variation among subjects or highly correlated with other features using the “findCorrelation” function in the “caret” package (Kuhn, 2008), version “6.0-78.” The “findCorrelation” evaluates the pair-wise correlation of features. Then, it finds the highest absolute pair-wise correlation, if two features have a high correlation (*r* ≥ 0.9 Pearson's correlation), it eliminates the feature with the highest mean absolute correlation. It should be noted that other feature selection methods could be used to select the best features using NCV approach. However, the interpretation of such approach could be challenging i.e., the selected features from the inner loop of the NCV may vary across folds. In addition, using other feature selections should be applied within each loop of NCV, which increases the computational overhead. Thus, removing correlated features provides a better way to select features in this case. Figures S2, S3 in Supplementary shows the correlation matrices before and after removing the correlated features.

### Machine learning methods

Selecting appropriate ML algorithms is a critical step to achieve robust BrainAGE estimation. Having represented each subject's features in one row, the final dataset dimension is *x* = *n*×*m*, with *n* = 468 and *m* = 863. We used R package “caret” to perform a set of regression algorithms: Elastic Net (ENET), Support Vector Regression (SVR), Random Forest (RF), extreme gradient boosting tree (XgbTree), and Gaussian Process with Polynomial Kernel (gaussprPoly). The aim is to test different ML techniques in order to provide a better estimation for age. First, ENEST is a linear regression technique that uses L1 and L2 regularization to prevent overfitting. Second, SVR uses optimization to build the regression model, but in high dimensional version of the training data. In our case, we used a kernel with radial basis function to project the data into high dimension space. Third, RF is one of the most common ensemble techniques, where it performs subsampling for the feature space of training data to build multi weak learners. Thus, different models from the training data are produced and then averaged to minimize the variance across models. Fourth, XgbTree utilizes a combination of ensemble learning, optimization and regularization to build generalized model from training data. Finally, gaussprPoly is a probabilistic approach to build a regression model by learning the distribution of the training data given the response (age). Similar to the kernel function in SVR, gaussprPoly adopts a polynomial kernel to project data into high dimension space.

**Figure 2:**
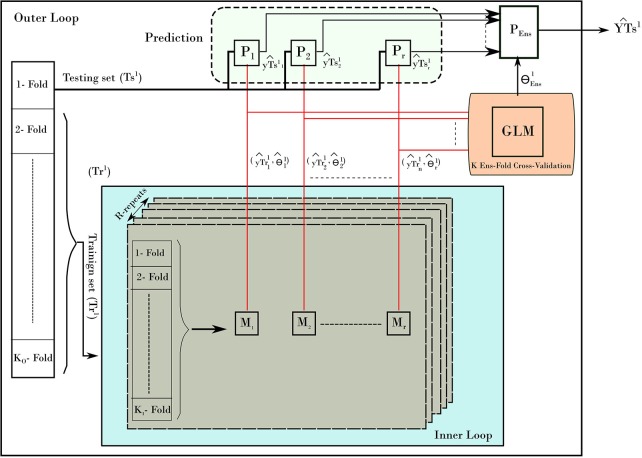
The nested-cross-validation procedure for predicting age. The example here demonstrates the first fold of the outer loop. The procedure consists of an inner loop (blue color) and outer loop. The inner loop is used to find the best models to predict the age. The outer loop uses those models to predict the age on the testing set. The process is repeated for all folds of the outer loop, which results in building a prediction of age from all samples.

To provide un-biased prediction for age, the nested cross validation was adopted in building age prediction models (Varma and Simon, 2006). Figure 2 depicts the NCV procedure consisting of two main loops: the inner and outer loops. The inner loop is used to find the best parameters from training set, while the outer loop is used to evaluate the best parameters on the testing set. To elaborate on the NCV, let the subscript refers to data and models from inner loop of NCV, while the superscript represents the ones from outer loop. In our run, we used 10-fold cross-validation (*K*_*I*_ = 10) for the inner and 10-fold cross-validation for outer loop (*K*_*O*_ = 10). The inner loop was used to estimate the best parameters on training data (*Tr*^1^) using a grid search and the one-standard error rule. Each inner loop consists of 5-repeat (*R* = 5) for each method. The outer loop uses the best obtained models to build a stack-ensemble model. The best models are represented by its best parameters${\;\theta }_{i}^{l}$, with *i* is the method index of the corresponding method *M*_*i*_ (*i* = 1.*r*) and *l* refers to the fold *l* from the outer loop. The symbol “*P*” refers to the prediction process associated with each method. Stacking ensemble helps to improve the stability of prediction by combining the prediction from other models; i.e., predictions from the five methods were combined by learning weights via a general linear model (GLM). In details, the GLM was trained on the resampled predicted age from the inner loop ($yTr_{i}^{l}$). Then, the GLM was used to provide one weighted-average prediction in 10-fold cross-validation (*K*_*Ens*_ = 10). From there, the best stack-ensemble model ($\theta_{Ens}^{l}$) was used to predict age for the testing set ($\hat{YTs^{l}}$). That is, the prediction of age is calculated for the individual methods${\;yTr}_{i}^{l}=P_{i}\left(Tr^{l},\;\theta_{i}\right)$, and then the weighted average is estimated for fold *l*.

$$\begin{array}{l} \hat{YTs^{l}}=P_{Ens}\left(\left[yTr_{1}^{l},yTr_{2}^{l},\;\ldots ,\;yTr_{r}^{l}\right],\;{\;\theta }_{Ens}^{l}\right) \end{array}$$

**Figure 3:**
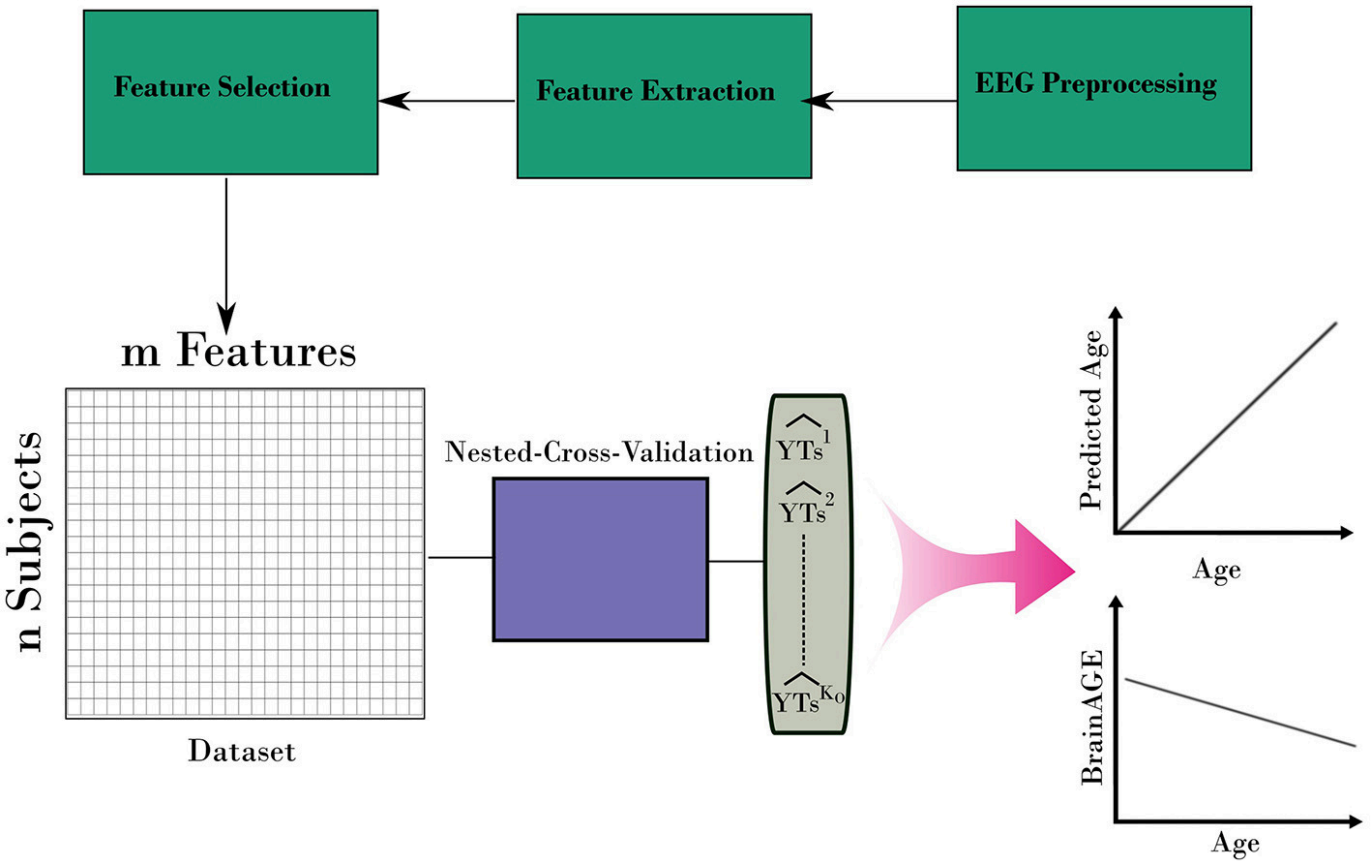
The complete framework for estimating the BrainAGE form EEG. The framework uses the nested-cross-validation method (Figure 2) to build estimation for the age. Then, those estimations are used to calculate the BrainAGE from the entire dataset.

After iterating over all folds from the outer loop, a prediction for the age for the entire dataset can be built. In addition, the variable importance of predictors from the stacking ensemble models was estimated across the outer loop of NCV. Finally, the predicted age and age values were used to estimate the BrainAGE for the dataset. Figure 3 shows the overall framework to estimate the BrainAGE.

## Results

**Figure 4:**
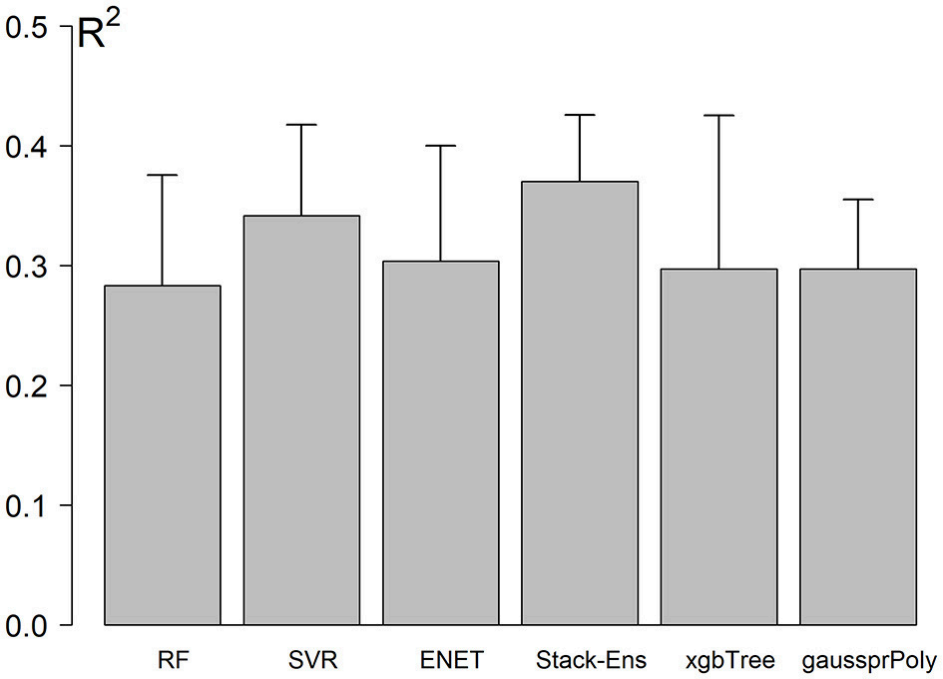
Models performance using NCV. Error bar represents the standard deviation of performances across the outer loop of NCV.

The NCV R-Squared performance for Stack-Ensemble and underlay methods is shown in Figure 4. The individual performance for each ML method was calculated before the stack-ensemble phase. The results showed that SVR with radial kernel achieved the best accuracy *R*^2^ = 0.34(0.056), MAE = 7.01(0.68) years and RMSE = 8.7(0.63) years. On the other hand, the stack-ensemble improved the overall performance with *R*^2^ = 0.37 (0.064), MAE = 6.87(0.69) years, and *RMSE* = 8.46 (0.59) years.

**Figure 5:**
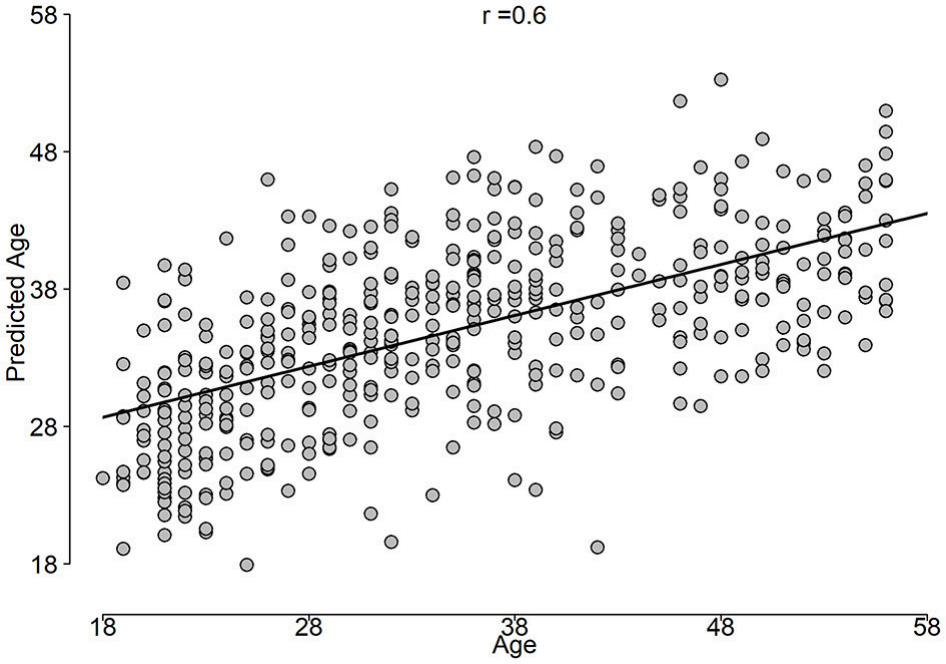
Predicted age vs. age constructed from the outer loop of NCV.

**Figure 6:**
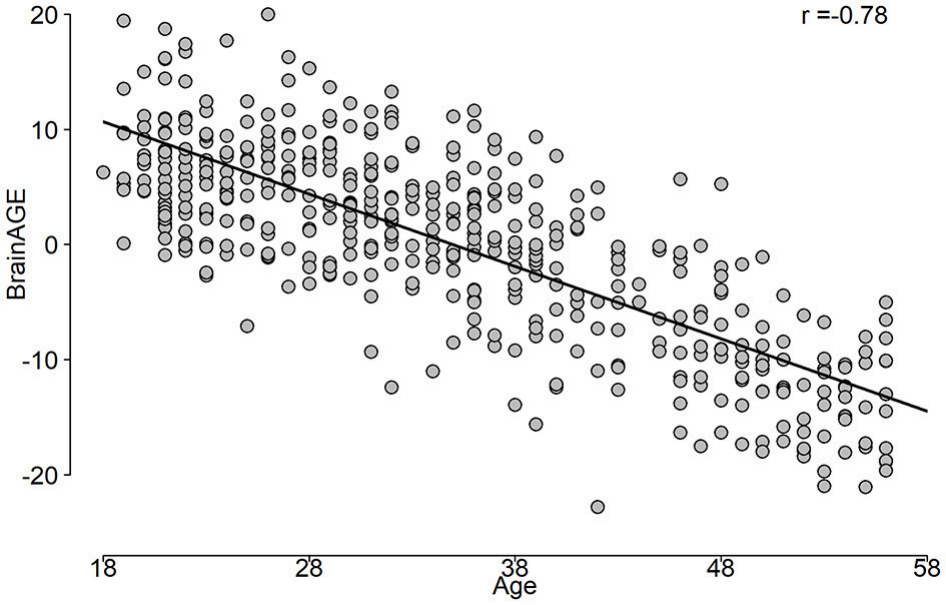
The BrainAGE variable as a function of the chronological age.

The correlation between predicted age and age is shown in Figure 5, while the BrainAGE variable is plotted in Figure 6.

**Figure 7:**
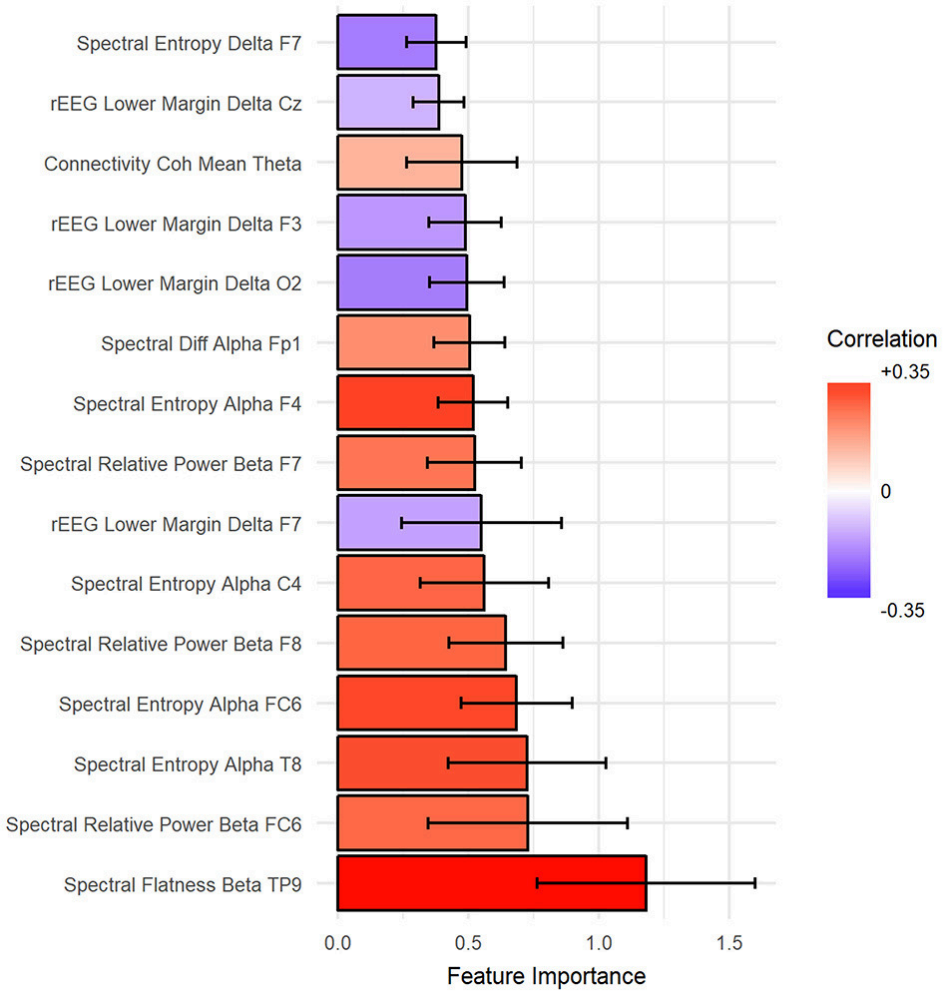
The top 15 important features to predict age sorted from most important (bottom) to top. Ventricle axis shows the scoring values from stack-ensemble model predictor, while the color indicates the correlation values between that feature and age.

The importance of features was estimated such that the total summation of features importance is 100 from each fold of the outer loop of NCV. Then, the importance scores were averaged across folds. In our case, we report the results as the mean across all folds. Figure 7 shows the top 15 important predictors of age. The color of the bars represents the Pearson's correlation values between each predictor and the age. From the graph, we can notice that “spectral flatness of beta band from channel TP9” is the most important predictor of age with *r* = 0.34. Please refer to Figure S1 in Supplementary for detailed graphing for the relationship between top predictors and age.

**Figure 8:**
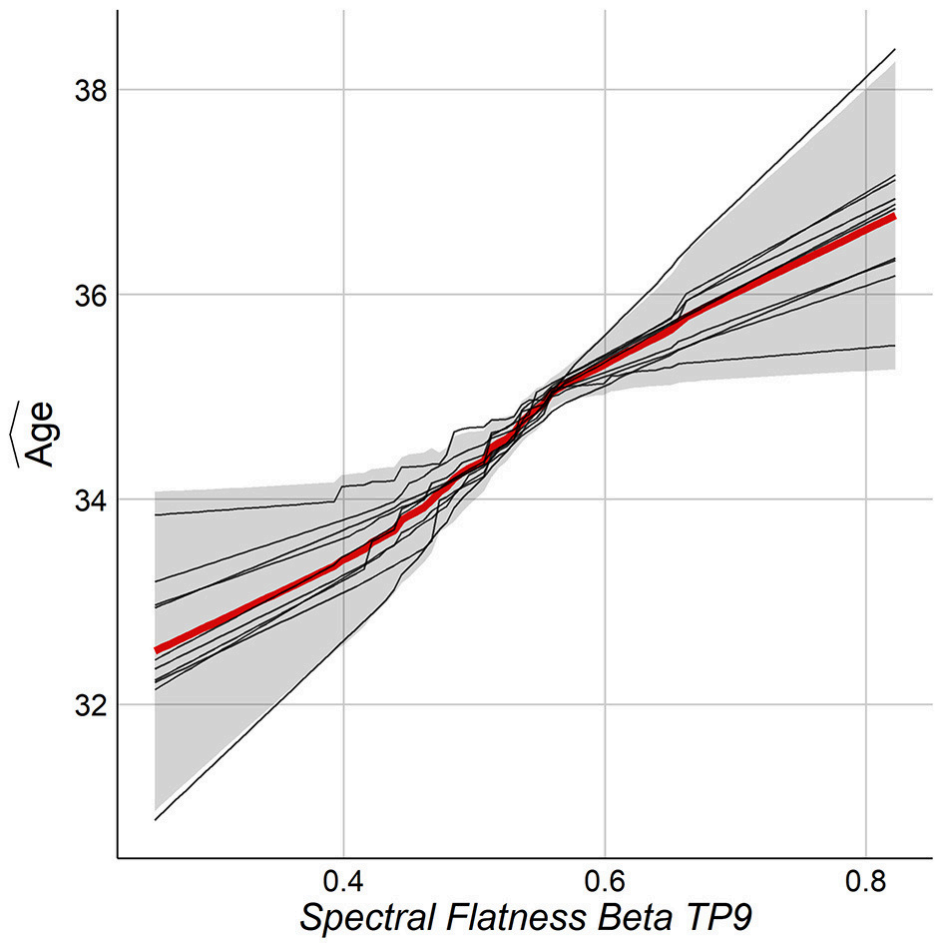
PDP for the top feature from NCV from Stack-Ensemble model.

The relationship between chronological age and the top features was studied by the Partial Dependence Plot (PDP) (Friedman et al., 2001). For each training model, the consistency across folds was examined by overlaying the PDP curves. One wants the same feature to behave similarly among the folds of the outer loop of NCV. Figure 8 shows the PDP for the top feature. As can be seen, the PDP for each fold (thin lines) have consistent behavior among all folds. Figure S1 in Supplementary shows PDPs for the top features.

**Figure 9:**
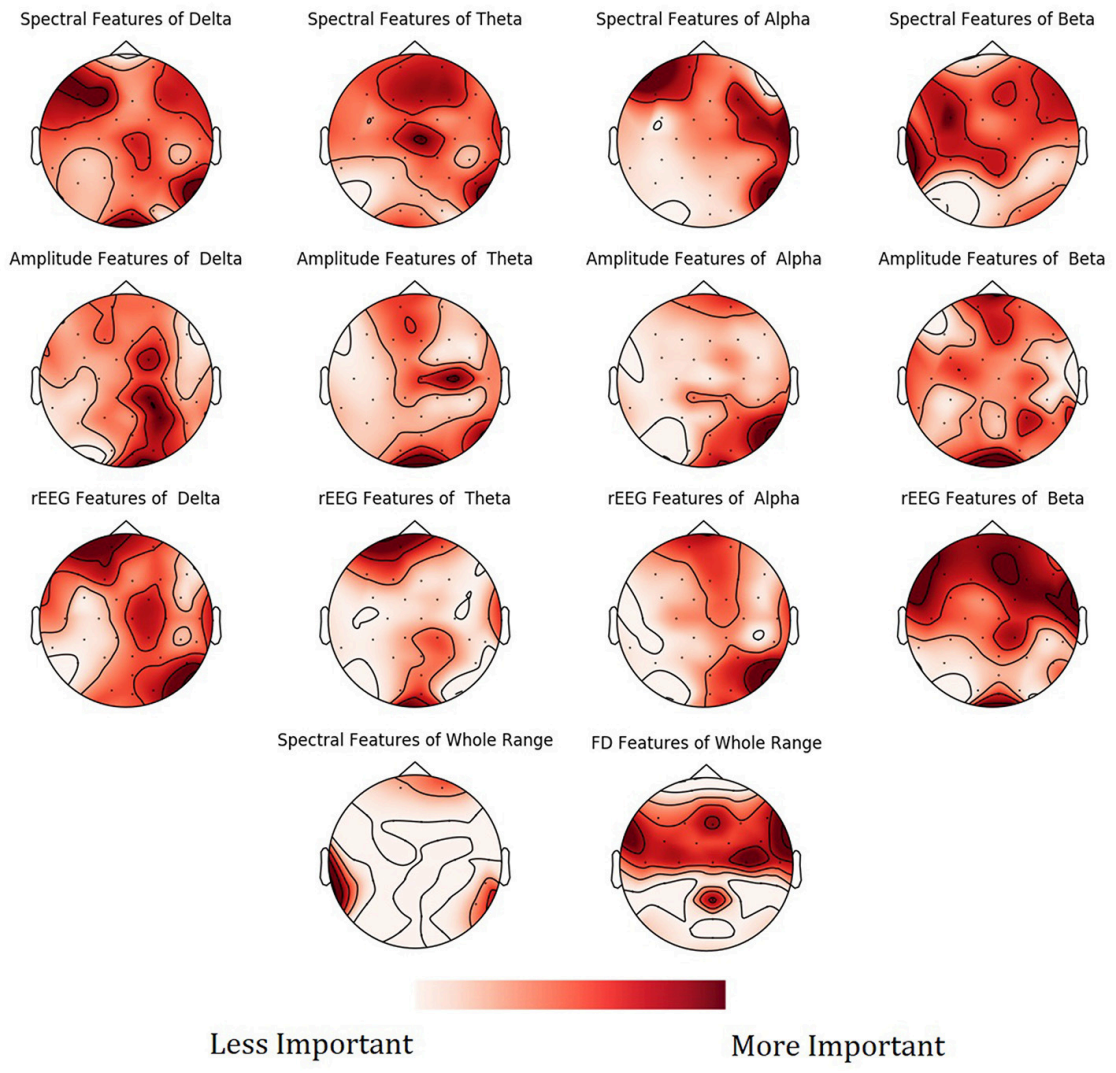
Mean feature importance scores sorted by bands and channels for predicting Age. The darker the color, the more important is the feature.

To show the spatial distribution of feature importance, MNE software (Gramfort et al., 2013) was used. More specifically, the feature importance scores obtained from the NCV were averaged based on the feature type and categorized based on the frequency bands. The resultant mapping for the feature importance scores is shown in Figure 9. Figure S1 in Supplementary presents the PDPs for the top features.

**Figure 10:**
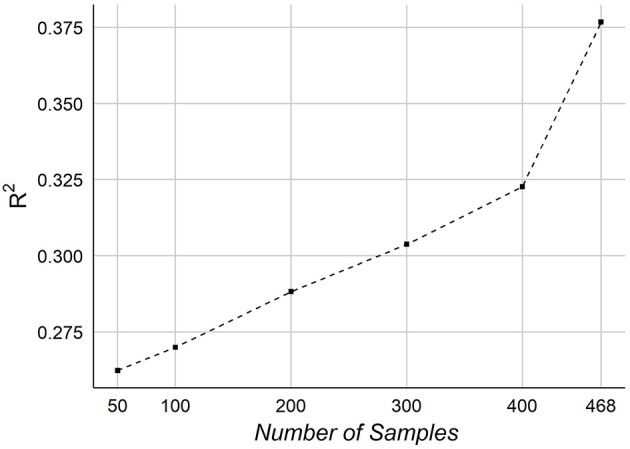
The effect of the number of samples on the age prediction.

Finally, we consider the effect of number of samples on the performance of predicting age. We tested our framework on different number of samples. Figure 10 graphs the *R*^2^ of NCV as a function of the number of samples in our dataset.

## Discussion

In the discussion part, we address the results, our research goals and elaborate on different implementation details. In addition, we compare our results with related work and point out various aspects of differences.

### Age-related changes are affecting brain EEG signals

Results suggest that indeed the aging affects human brain EEG signals. We have also determined that, a comprehensive feature extraction is required from EEG signals to capture the relationship between chronological ge and the age predictors. This suggests that the aging is reflected broadly on the EEG signals without selected predominate feature and also suggests that utilized EEG predictors feature different mechanisms of influence by age and/or disease. In addition for features extraction, selecting the best features is important to improve the performance and reduce the complexity of the model. We eliminated the correlated features to select the best features, which improve the overall *R*^2^. Our selection for correlated features preserves the consistency among NCV folds and importantly eases the interpretation of the results. The age-related changes in EEG are strongly supported by the literature (Benninger et al., 1984; Gasser et al., 1988; Marciani et al., 1994; Widagdo et al., 1998; Kikuchi et al., 2000; Babiloni et al., 2006; Hashemi et al., 2016) and by our results as well, where the correlation between top four features and age was relative high with *r* = 0.34, 0.3, 0.26, and 0.24, respectively.

### Can age be predicted from EEG signals?

Using unbiased prediction of age, NCV, we were able to provide a reasonable accuracy for predicting age. The best results were obtained by SVR (*R*^2^ = 0.34) and were slightly improved by the Stack-ensemble approach (*R*^2^ = 0.37). The correlation between predicted age and age (*r* = 0.6), which shows the ability of our model to predict the age. The overall feature importance scores were extracted for each fold in the outer loop of NCV and then averaged across all folds. The feature importance showed that the important predictors are spread out across different features types and bands. In addition, we used PDP to examine the consistency of features across the outer loops of NCV, where we showed that top features have a similar behavior across the folds.

The effect of the number of samples on prediction accuracy is shown in Figure 10. The graph indicates a potential improvement may be achievable adding more samples. When testing on 50 samples, the overall accuracy was *R*^2^~ = 0.26, which shows that the features are informative for predicting age even from small number of samples. It should be noted that our samples size is relatively smaller than other works, especially those ones used MRI.

We found no differences in age prediction across female and male groups. Both groups have a relatively matched average chronological age: female group = 34.47 (10.65) and male group = 35.29(10.47). The average predicted age resulted in 34.78 (6.87) for female and male 35.11 (6.04). The MAE was 6.99 (5.10) and 6.66(4.77) years for female and male groups, respectively.

Mapping of the spatial distribution of feature importance scores revealed that age predictors are not uniquely corresponding to specific channels, frequency band nor to a specific feature domain. That is, different features types capture some characteristics of EEG, but not the whole relationship. For example, Figure 7 showed that among the top 15 important features, the spectral features are positively correlated with age, while rEEG features are negatively correlated. That is, one type of features captures a specific aspect of the relationship between that feature type and the age. Thus, providing heterogeneous features can improve the predictability of age. This is also supported by Figure 9, where the spatial distribution of feature importance scores does not exhibit a uniform representation. Our analysis shows that relative contribution of features importance is 46, 31, 18, 3, and 2% for spectral, rEEG, amplitude, FD, and connectivity, respectively. It should be noted that the number of features among different domains are not the same especially that is the case for FD and connectivity features. Similarly, features contributions are also spread out across bands as follows: 31, 21, 27, and 18% for theta, delta, alpha, beta, and theta, respectively.

### Comparison with other works

Predicting age from EEG features was also studied in Dimitriadis and Salis (2017). Compared with the current study, they reported relatively higher prediction accuracy, 0.6, compared with 0.4 here. There are a number of differences which may contribute to this disparity. Perhaps the most significant one is that they seem to have done feature selection using the response variable and the entire dataset, which will generally lead to more optimistic evaluations than doing feature selection within a nested cross validation framework, as done here. Additionally, we report *R*^2^ as 1-SSresid/SStotal (SSresid is the squared residuals from the regression and SStotal is the total sum of squares of differences from the mean) taken from the model prediction, while they seem to have reported the *R*^2^ of a line fit through Age vs. Predicted Age. Other differences include the feature sets used and the fact that our data were collected during fMRI, which may leave some residual artifact. Furthermore, we use here an interpretation-friendly features.

Predicting age from functional brain imaging is probably more challenging than structural imaging. One can notice from Table 1 that fMRI yields generally a lower performance than MRI data. The best results was reported by Cole et al. (2017) with *r* = 0.96 from structural imaging of healthy subjects. EEG and fMRI are both functional imaging for the brain and thus it's more subjective to compare EEG results with fMRI results. Our method's performance is relatively lower than those from fMRI works reported in Dosenbach et al. (2010) with *R*^2^ = 0.55 and Qin et al. (2015) with MAE = 4.6 years. Without a subjective comparison between EEG and fMRI from the same dataset, it's hard to draw conclusions about amount of information that each domain embeds. Although fMRI/MRI imaging may yield a higher accuracy, but it comes at extra cost and less portability as compared to EEG.

The contribution of some features in BrainAGE is in line with previous works (Chiang et al., 2011; Zappasodi et al., 2015). For instance, our findings show the negative correlation between age and alpha power spectra in healthy groups reported in Chiang et al. (2011). This correlation trend could be observed in other frequency bands, especially Delta and Theta bands. FD is positively correlated with age for Healthy subjects, which is consistent with finding in Zappasodi et al. (2015). However, Zappasodi et al. (2015) showed that FD increases for ages from 20 to 50 years and then decreases. Since our age limit is 58, the pattern is increasing overall for ranges from 18 to 58 years. Figures S6, S7 in Supplementary provide a spatial mapping of the correlation values between the spectral and FD features and age.

## Conclusions

We have introduced the rigorous framework for BrainAGE estimation based on EEG brain signals. Proof-of-concept analysis showed that, it is possible to build a robust BrainAge estimation by harnessing both extensive EEG feature representation and suitable ML algorithms. ML and NCV play a significant role in identifying informative features and studying the spatial distribution of significant predictors, and providing unbiased prediction. In addition, we showed how to evaluate and interpret the results using the feature importance scores and partial dependence plots. The introduced framework can be extended to test association with and predict other physiological relevant measures based on EEG brain signals.

## Author contributions

All authors contributed significantly to work regarding conception and design; acquisition, analysis; drafting the work and revised critically; approval of the version to be published, and carrying the responsibility for achieving the accuracy or integrity of any part of the work.

### Conflict of interest statement

The authors declare that the research was conducted in the absence of any commercial or financial relationships that could be construed as a potential conflict of interest.
