# Supplementary material for: Predicting Age From Brain EEG Signals—A Machine Learning Approach
Source: Front Aging Neurosci. 2018 Jul 2;10:184. doi: 10.3389/fnagi.2018.00184 (PMC6036180; doi:10.3389/fnagi.2018.00184)
Supplement: Supplementary file 1 [file Data_Sheet_1.DOCX]

Supplementary Material

**Predicting age from brain EEG signals – a machine learning approach.**

**Obada Al Zoubi^1,2^, Chung Ki Wong^1^, Rayus Kuplicki^1^, Henry Yeah^1^, Ahmad Mayeli^1,2^, Hazem Refai^2^ , Martin Paulus^1^ and Jerzy Bodurka^1,3^**

# Supplementary Figures and Tables


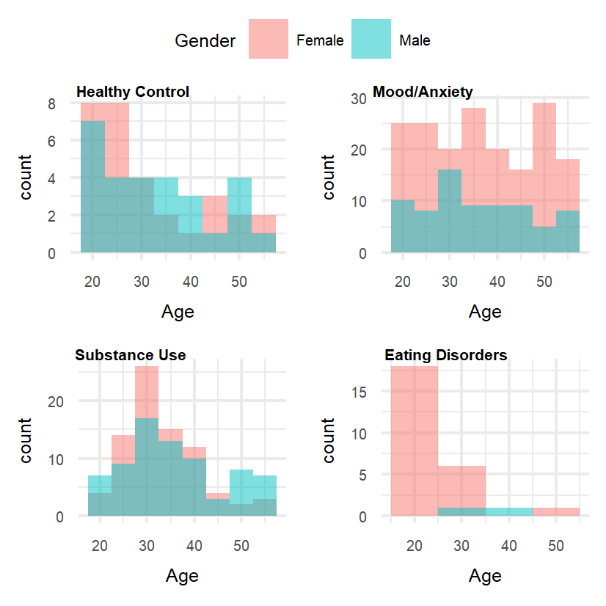


Figure 1. Histogram for the dataset divided based on groups and gender.


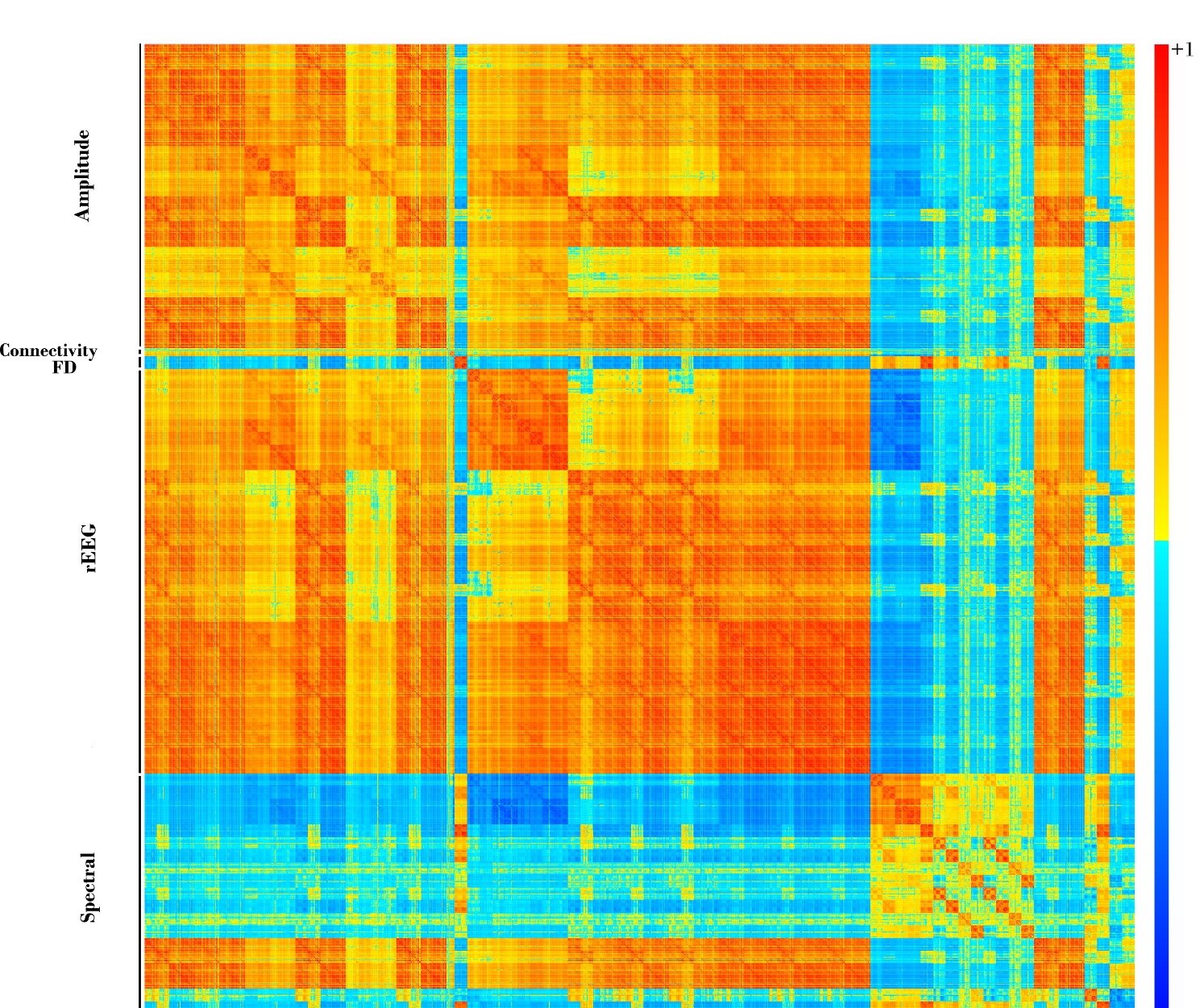


Figure S2. Correlation matrix for all features (before removing correlated features) arranged based feature type.


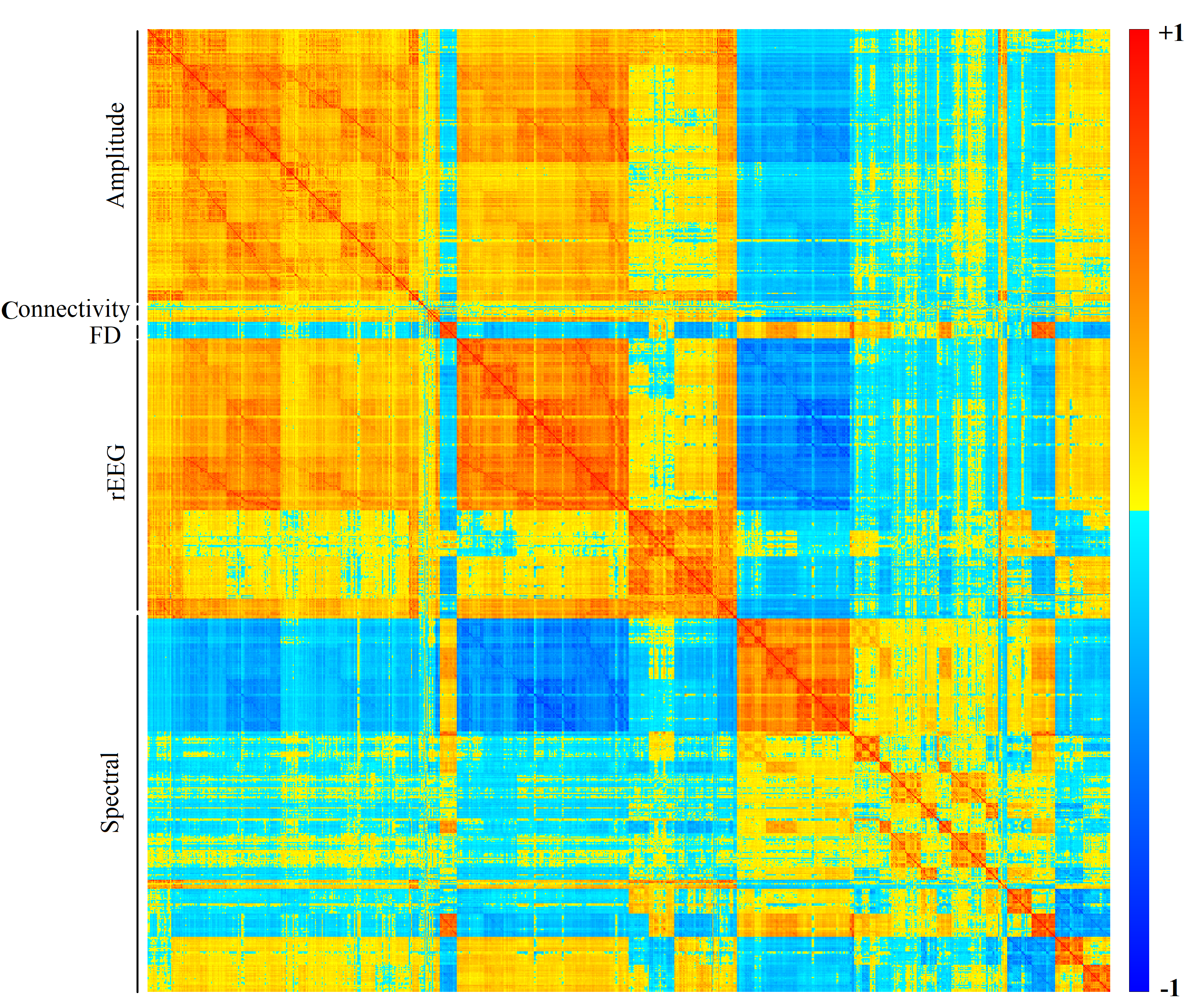


Figure S3. Correlation matrix for features (after removing correlated features: Thr=0.9) arranged based feature type.

| 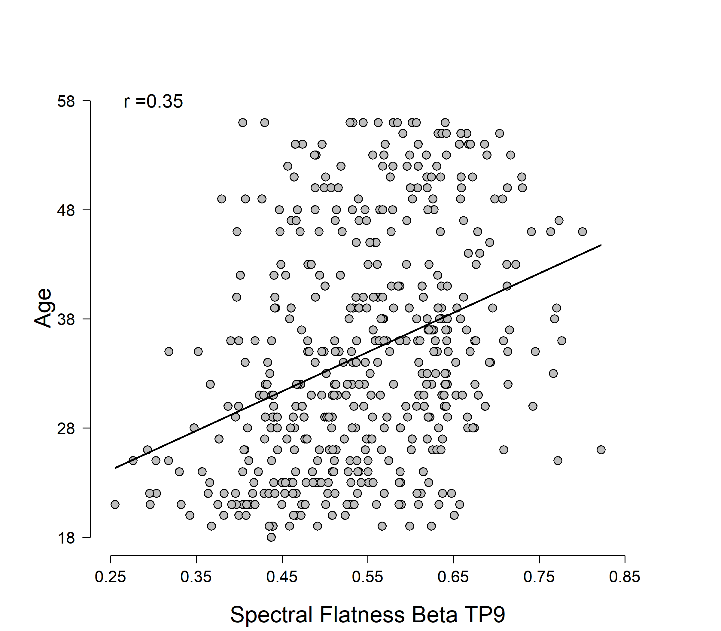 | 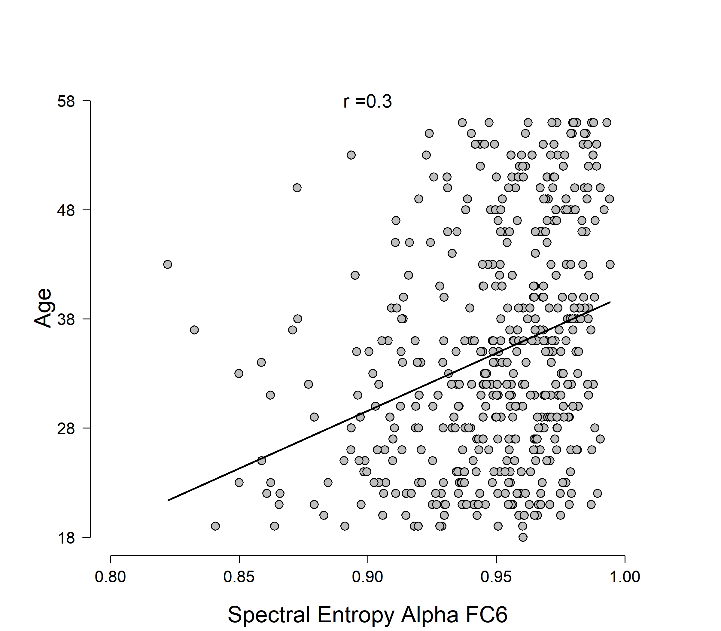 |
| --- | --- |
| 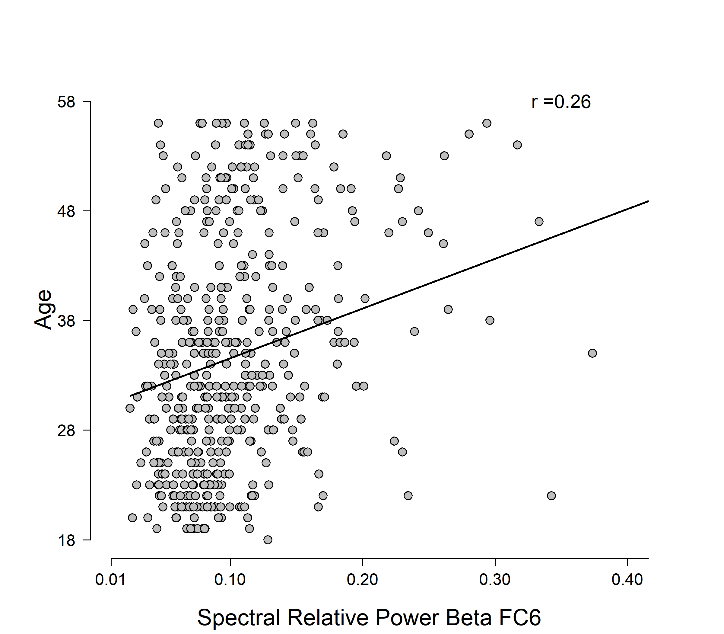 | 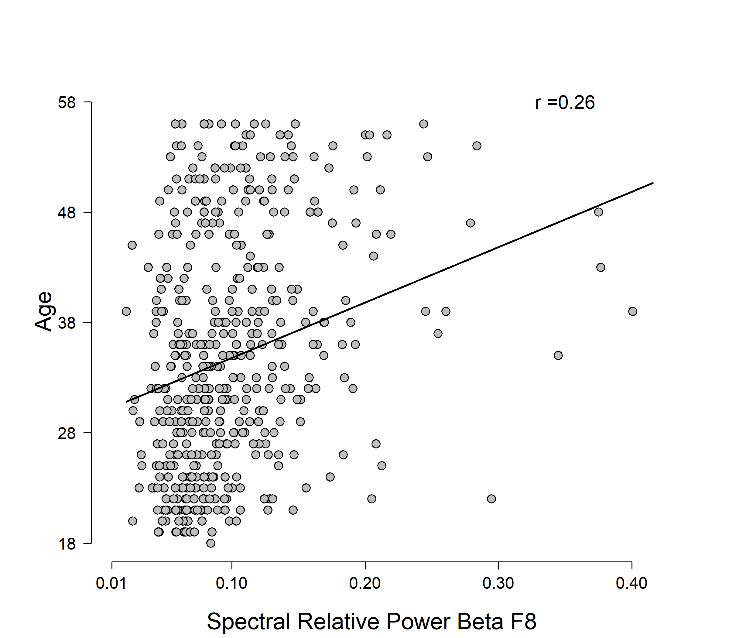 |

Figure S4: Top features correlation with age.

| 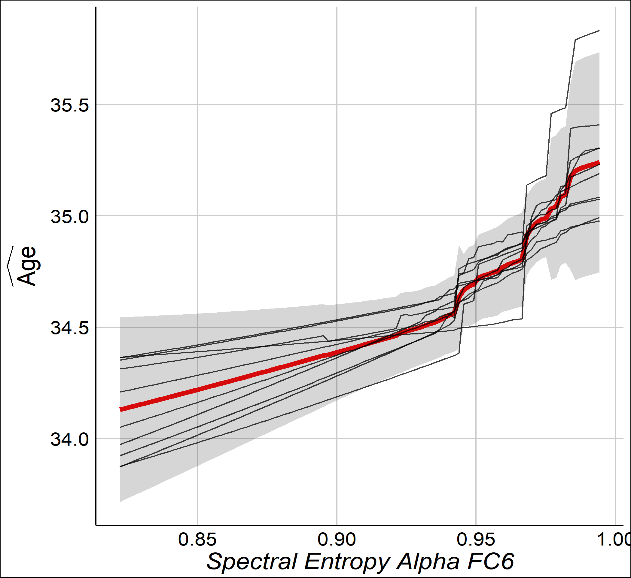 | 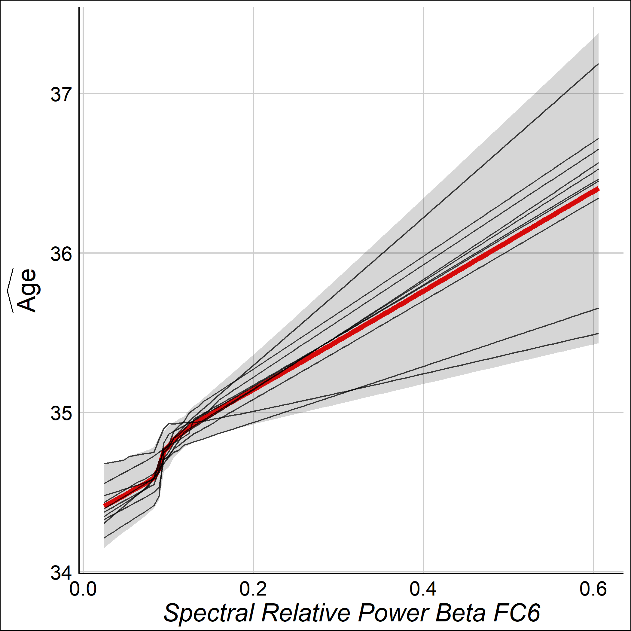 |
| --- | --- |
| 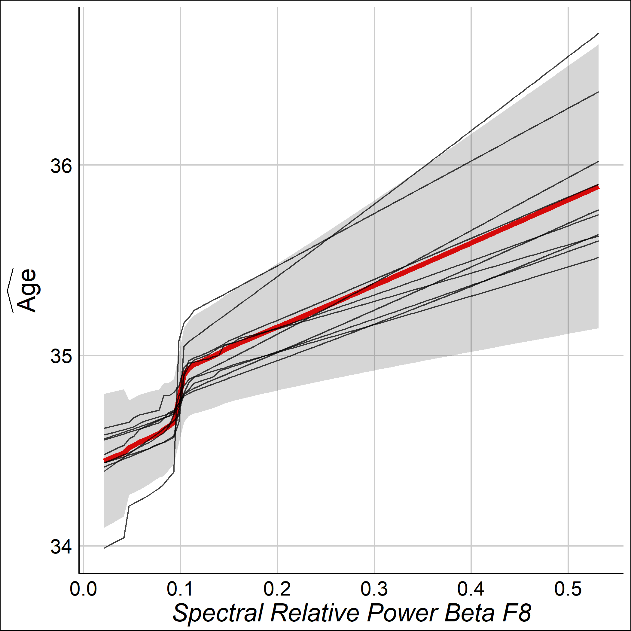 | 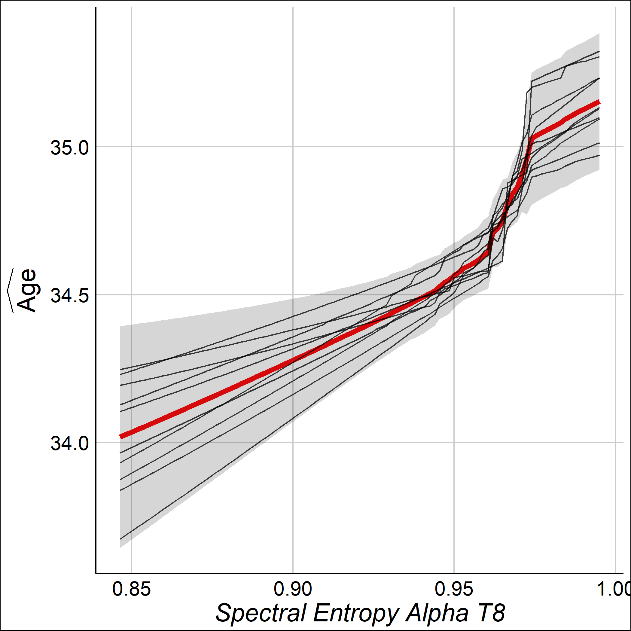 |

Figure S5: The PDPs from Stack-Ensemble model for the top features. The red line represents the average values over the folds of NCV, while the thin lines are the individual PDPs for each fold.


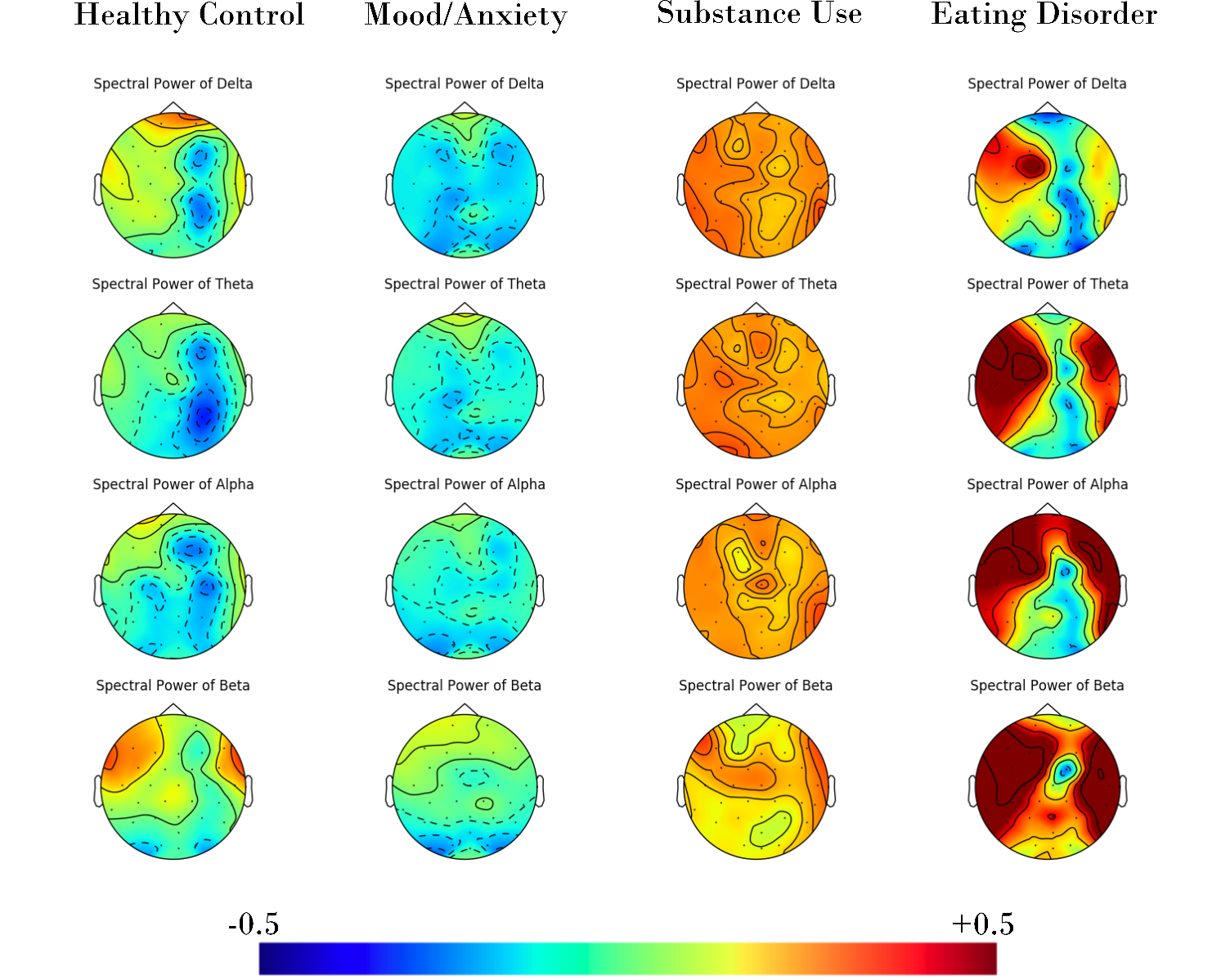


Figure S6. The correlation between spectral power feature and chronic age across different frequency bands and groups.


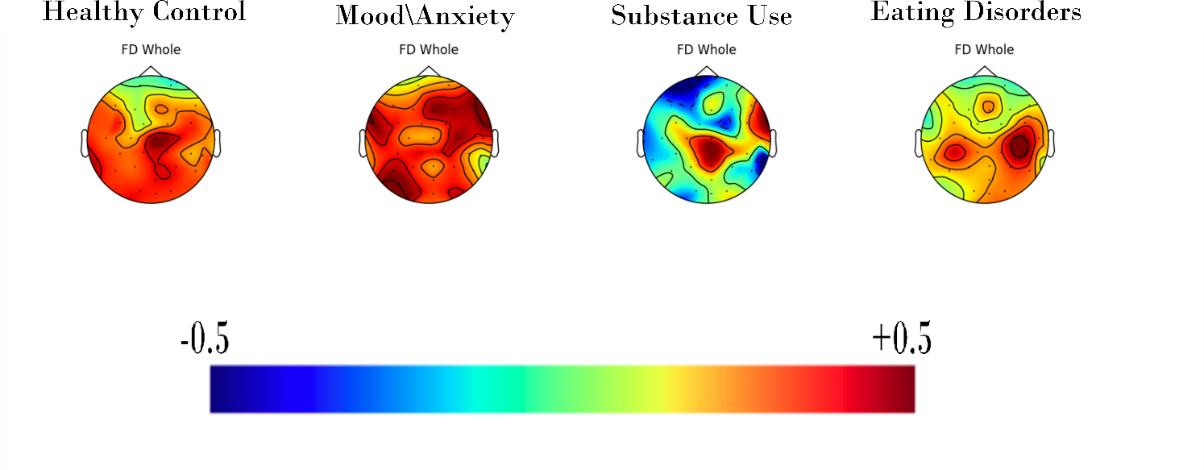


Figure S7. The correlation between FD feature and chronic age across groups (for the whole EEG frequency).
